# Correlation set analysis: detecting active regulators in disease populations using prior causal knowledge

**DOI:** 10.1186/1471-2105-13-46

**Published:** 2012-03-23

**Authors:** Chia-Ling Huang, John Lamb, Leonid Chindelevitch, Jarek Kostrowicki, Justin Guinney, Charles DeLisi, Daniel Ziemek

**Affiliations:** 1 Bioinformatics Graduate Program, and Department of Biomedical Engineering, Boston University, 44 Cummington Street, Boston, MA 02215, USA; 2 Computational Sciences Center of Emphasis, Worldwide Research & Development, Pfizer, 35 Cambridgepark Drive, Cambridge, MA 02140, USA; 3 Oncology Research Unit, Worldwide Research & Development, Pfizer, 10646 Science center Drive, San Diego, CA 92121, USA; 4 Sage Bionetworks, 1100 Fairview Ave North, Seattle, WA 98109, USA

## Abstract

**Background:**

Identification of active causal regulators is a crucial problem in understanding mechanism of diseases or finding drug targets. Methods that infer causal regulators directly from primary data have been proposed and successfully validated in some cases. These methods necessarily require very large sample sizes or a mix of different data types. Recent studies have shown that prior biological knowledge can successfully boost a method's ability to find regulators.

**Results:**

We present a simple data-driven method, Correlation Set Analysis (CSA), for comprehensively detecting active regulators in disease populations by integrating co-expression analysis and a specific type of literature-derived causal relationships. Instead of investigating the co-expression level between regulators and their regulatees, we focus on coherence of regulatees of a regulator. Using simulated datasets we show that our method performs very well at recovering even weak regulatory relationships with a low false discovery rate. Using three separate real biological datasets we were able to recover well known and as yet undescribed, active regulators for each disease population. The results are represented as a rank-ordered list of regulators, and reveals both single and higher-order regulatory relationships.

**Conclusions:**

CSA is an intuitive data-driven way of selecting directed perturbation experiments that are relevant to a disease population of interest and represent a starting point for further investigation. Our findings demonstrate that combining co-expression analysis on regulatee sets with a literature-derived network can successfully identify causal regulators and help develop possible hypothesis to explain disease progression.

## Background

Fundamental functions of living cells are controlled by regulatory relations between genes and proteins. Most cell types respond to changes in their environment (e.g. drug treatments or disease causing mutations) by altering their transcriptional patterns. More than a decade ago, it became possible to measure snapshots of all transcript levels in a given tissue sample using microarray technology. Since then, advances in technology have multiplied and the cost of experiments has decreased significantly. As a consequence, cell lines, animal models as well as clinical subjects in drug trials or in the general population [1] have been characterized on a molecular level. One crucial problem in such studies is the detection of *active *key regulators; i.e. genes or proteins that *causally *affect expression of downstream genes or proteins in the study population.

The detection of regulators or regulatory networks from the primary data alone has been studied extensively. Network reconstruction can be approached by identifying correlations in expressed genes [2], using any number of methods including those based on information theory [3], Bayesian models [4,5] and regression models [6]. However, all purely expression-based methods assume that the expression of regulators and targets are directly (anti-) correlated or at least not independent of each other. Smet *et al. *showed that such models of high correlation between regulator and target don't match the actual situation captured in RegulonDB [7]. One of the possible explanations is that a regulator, even a transcription factor, itself is not necessarily co-expressed with its targets, especially when the regulatory relation of the regulator and the targets is complex [8] or regulation acts beyond the transcriptional level, e.g. by phosphorylation. This suggests that in many cases the activity of regulator cannot be inferred from transcriptional data alone.

While methods to infer causal regulators directly from heterogeneous primary data types have been proposed and successfully validated in some cases [9,10], these methods necessarily require very large sample sizes and a mix of different data types, e.g. genomic and transcriptomic data. Even when such large data sets are available, the choice of which hypothesis to pursue in follow-up experiments might not be easy to make as such methods usually don't relate their conclusions back to already known biological facts.

In this paper we are interested in a method that suggests active regulators and corresponding perturbation experiments for a population based on expression data alone. With that goal in mind the above discussion suggests that investigating coherence between regulatee pairs rather than regulator-regulatee pairs might be more suitable for evaluating the activity of a given regulator. To associate regulatee coherence to a possibly only non-transcriptionally controlled regulator requires the use of prior knowledge.

Our knowledge of molecular biology has increased dramatically over the past several decades as evidenced by the 20 million articles currently indexed by the National Library of Medicine [11]. This immense body of knowledge contains many experiments that define the response of a biological system to a stimulus, e.g. by altering its transcriptional state. These experiments can be translated into causal regulatory relationships. Whenever the question of causal regulators is relevant (e.g. in finding potential intervention points in ovarian cancer), each experimentally validated finding constitutes a hypothesis that can be evaluated based on the data set at hand.

In contrast to many previous approaches, we rely on a very specific type of prior knowledge, namely relationships extracted from the literature that are (a) based on well-described laboratory experiments, (b) have a citation in the literature, and (c) establish a flow of causality; i.e. a directed flow of information form a well-defined perturbation experiment to observed molecular changes. Consequently, our method does not rely on association but on established causation and should be well-suited to providing hypotheses about the causal regulators that are active in a given dataset. This specifically allows us to make statements about regulators that are not limited to activation by changes in transcript abundance, but can include changes in protein abundance or post-translational modifications.

Prior biological knowledge has been used successfully in many contexts before. Relevant functional terms, dysregulated pathways in diseases as well as active miRNAs and transcription factors are routinely predicted based on so-called Gene Set Analysis (GSA) methods and a number of statistical procedures have been proposed, e.g. [12-16]. However, virtually all such methods focus on differential gene expression between *two *conditions as opposed to coordinated changes in a subject population. The situation is similar for methods that utilize networks of prior information [17,18]. A class of methods that has been proposed to detect subnetworks co-expressed across a population [19,20] is related in that it utilizes expression data across all conditions in conjunction with a network. However, such methods aim at finding coherence between expression correlation and distance in the network in general. Our goal is to specifically assess whether a regulator is likely to be active based a given expression data set and consequently point the researcher to relevant perturbation experiments in the literature.

To this end, we introduce the *Correlation Set Analysis *(henceforth referred to as CSA) method in the following, provide evidence that it performs well on simulated datasets, and apply it to three different disease settings: ovarian cancer [21], metabolic disease [22], and diffuse large B-cell lymphoma (DLBCL) [23].

## Methods

**Figure 1:**
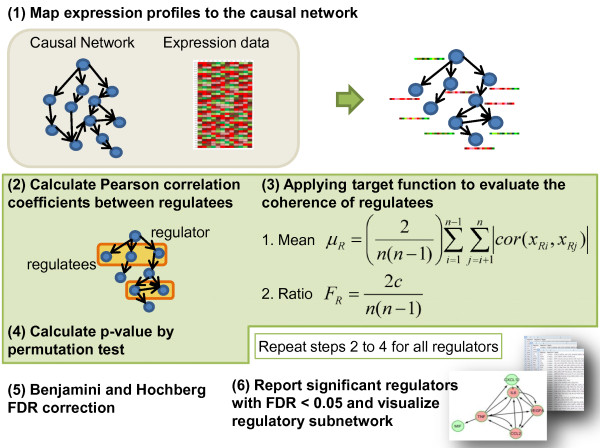
**Schematic illustration of the Correlation Set Analysis (CSA) method**. Details are described in the text.

We identify regulators that are active under a given condition if (a fraction α of) its known targets show (a specified degree β of) correlated transcriptional change. This method does not infer novel regulatory relationships, but rather selects from a library of experimentally defined relationships which regulators test as active in the population of interest. Figure 1 provides an overview of CSA. We elaborate on each step below.

### Constructing the causal network

The suggested method relies on a causal network to define regulators and regulatees and can only be as good as the encoded biological facts. The causal network consists of relationships that (a) are based on well-described laboratory experiments, (b) have a citation in the literature, and (c) most importantly, establish the flow of causality directed from a specific regulator to a specific regulatee. Consequently, our method does not rely only on association, but on established causation. For example, consider the following statements extracted from two articles represented in the Ingenuity [24] data:

1. "Binding of mouse Fyn protein and mouse Cnr1 (Pcdha4) protein occurs in mouse brain." (PMID 9655502)

2. "Blockade of CB1 (CNR1) increases expression of hepatic lipase (LIPC)." (PMID 20110567)

While statement 1 asserts a biologically correct binding event, it doesn't imply a directed flow of information and it is unclear what consequences the binding event has. In contrast, statement 2 describes a perturbation experiment that causally leads to observed changes. Only statement 2 allows for a meaningful definition of regulator and regulatee. Ultimately, the use of causal statements facilitates the interpretation of results and focuses the analysis on potential upstream drivers of the process under consideration.

Regulators and regulatees can be of different molecular types and include transcript levels, protein levels, protein activities and phosphorylation states. For the purpose of this method, we restrict our causal network to transcript regulatees as this is consistent with the population measures analyzed. In contrast, regulators include transcript and protein levels as well as protein modifications and activities. For CSA, we abstract these different forms into an undifferentiated node in the causal network based on their Entrez identifier [25].

It is important to note that the results of such transcriptional perturbation experiments do not necessarily capture *direct *physical relationships. In the example above, the increase in transcript levels of LIPC is certainly mediated by a cascade of other signalling molecules. Consequently, the CSA method is not limited to transcription factors as regulators, but encompasses many other classes of molecules amenable to perturbation experiments.

To ensure the reliability of the data, we only include manually curated statements. The substrate for the causal network is licensed from two commercial sources, Selventa Inc. [26] and Ingenuity Inc. [24] and, after filtering and post-processing, reduces to 6,942 regulators and 11,134 regulatees. Among 6,942 regulators, 3,002 are proteins or mRNAs and 3,940 are chemical compounds or environmental factors (e.g. internal metabolites such as *glucose *or *pyruvate*, approved drugs such as *Rosiglitazone *or *Doxorubicin*, or environmental conditions such as *hypoxia *or *oxidative stress*). As described above from this we selected the subset of proteins and mRNA regulators. After removing self-regulation and regulators that only have one regulatee, the causal network reduces to 1,783 regulators and 10,097 regulatees. Selventa Inc. has recently launched an initiative to provide access to a significant amount of causal information to academic researchers through the BEL-Portal http://www.belportal.org. After performing similar pre-processing as we described above, the public causal network contains 823 regulators and 6,463 regulatees.

### Scoring putative regulators

We assumed an active regulator under a given condition should activate or inhibit a subset of its regulatees. Across a set of conditions (e.g. in a patient population), this relationship should become apparent in a coordinated change in expression levels for regulatees downstream of an active regulator. We used different scoring functions to identify active regulators.

#### Mean scoring function

Pearson's correlation coefficient is one of the most widely used measures to evaluate similarities of gene expression profiles. For an expression dataset with *m *samples, the co-expression level of any two genes *X *and *Y *can be calculated by the correlation coefficient *cor(X, Y)*.

$$cor\left(X,Y\right)=\frac{\overset{m}{\underset{k=1}{\sum }}\left(X_{k}-\bar{X}\right)\left(Y_{k}-Ȳ\right)}{\sqrt{\overset{m}{\underset{k=1}{\sum }}{\left(X_{k}-\bar{X}\right)}^{2}\overset{m}{\underset{k=1}{\sum }}{\left(Y_{k}-Ȳ\right)}^{2}}}$$

, where $\bar{X}$ and $\bar{Y}$ are sample means of gene X and gene Y respectively.

To assess the expression coherence of regulatee sets, we employed the simple test of measuring all pair-wise correlations within each set. Such a coherent regulatee set is consistent with the hypothesis that the corresponding regulator is active in the condition under consideration.

The causal network also specifies the type of regulation (i.e. up-regulation or down-regulation). Consider two regulatees, *X *and *Y*, that are under the control of a common regulator. If *X *and *Y *are regulated coherently, we expect their transcriptional profiles to be correlated. Conversely, if *X *is up-regulated and *Y *is down-regulated, we expect their profiles to be anti-correlated. We examined the correlation coefficients between up-regulated regulatees and down-regulated regulatees to test this hypothesis. However, we did not observe significant differences between correlation coefficients of regulatees regulated in the same direction and regulatees regulated in the opposite direction. Thus, we decided to use the absolute value of the correlation coefficient |*cor*| in the scoring functions.

One intuitive way of detecting regulators with highly coherent regulatee pairs is to examine the average of all absolute correlation coefficients between all pairs of regulatees *x_R _*for a regulator R.

$$\mu_{R}=\frac{2}{n\left(n-1\right)}\overset{n-1}{\underset{i=1}{\sum }}\overset{n}{\underset{j=i+1}{\sum }}\left|cor\left(x_{Ri},x_{Rj}\right)\right|$$

Here, *n *is the number of regulatees of the regulator R. *μ_R _*is referred to as the *mean *scoring function in the rest of this paper.

#### Ratio scoring function

**Figure 2:**
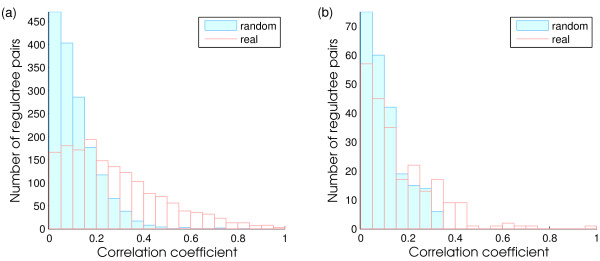
**The example distributions of absolute correlation coefficients between regulatees of a regulator detected by different target functions**. a) The average absolute correlation coefficient between regulatees in the real network (red) is significantly higher than it in the random network (blue). b) There is no significant difference between the absolute average correlation coefficients in the real network and the random network. However, there is a small bump at the right hand side, which means a small subset of highly correlated regulatees. The ratio scoring function was designed to detect such small subsets of regulatees.

If we expect that a substantial number of regulatees is affected by an active regulator, a test for a shift in mean pair-wise co-expression is sensible. However, we also investigated possible scenarios based on the biological data sets described in the results section. Figure 2a shows an example distribution of absolute correlation coefficients between regulatees which has higher average absolute correlation coefficients in a real network than in a randomized network. In this case, the majority of regulatees have similar expression patterns, which supports the hypothesis that this regulator is active. In some cases we observed a small bump at the high absolute correlation tail (Figure 2b), which indicates a small set of strongly co-expressed regulatees. This situation is more difficult to detect by examining the difference of average correlation coefficients. Hence, we propose an alternate way to detect active regulators: scoring regulators according to the ratio of highly coherent regulatee pairs over all regulatee pairs (referred to as the *ratio *scoring function).

$$F_{R}=\frac{2c}{n\left(n-1\right)}$$

, where *c *is the number of regulatee pairs, for a specified regulator *R *having *n *targets, with absolute correlation coefficient greater than a cutoff. Users can define biologically relevant pairs by setting the cutoff to levels appropriate to detect a desired effect size (say, correlation coefficient larger than 0.6). In this paper, we fix the cutoff, *c*, at the 95^th ^percentile of the distribution of all pair-wise correlations for a given dataset. This alternate score will identify small sets of highly coherent regulatees. The decision rule based on *μ_R _*and *F_R _*is described in the next section.

### Assessing statistical significance

These scoring functions provide rank-ordered lists of all regulators in the causal network based on the coherence of their downstream regulatees, and indicate which ones may be active. While true signals will tend to lead to high scores, high scores in any given result may be due to random noise. We therefore evaluated the statistical significance of the scores, *μ_R _*and *F_R _*of a regulator *R*, using a permutation test in two ways:

1. *Gene permutation *randomly assigns transcript profiles to regulatees and, thus, compares the score of the regulator *R *to the distribution of scores attained by regulators with the same number of randomly chosen regulatees.

2. *Graph permutation *generates a random causal network in which each regulator controls the same number of regulatees and each regulatee is controlled by the same number of regulators as in the original network. (Details are described in "Materials and Methods").

Both permutation approaches assess the statistical significance of a score under the respective null hypothesis, and thus provide guidance to the biologist as to whether a particular regulator received a high score based on chance alone. Note that the permutation of sample labels is not a meaningful option in the scenario of only one population when considering correlation though it is a preferred choice in many gene set analysis methods comparing two or more sample populations.

As the causal network contains more than one thousand potentially active regulators, the resulting p-values should be corrected for multiple testing. The false discovery rate (FDR) is an intuitive and well-accepted alternative measure of significance that is widely applied in similar applications. The Benjamini and Hochberg procedure was used to estimate the FDR based on the list of p-values [27]. Finally, CSA reports a results table of potentially active regulators (FDR < 0.05) which contains FDR, scores (*ratio *and *mean*), regulatees coherently up- or down-regulated by the regulator, non-coherently expressed regulatees, average correlation coefficient of regulator to regulatees, and the number of coherent regulatees. Users can rank regulators by the scores (*F_R _*or *μ_R_*), the number of coherent regulatees (*n_c_*), or the average correlation coefficient of regulator to regulatees (*μ_RR_*).

## Results and discussion

### Results on simulated data

To assess the sensitivity and specificity of CSA, we generated simulated data sets with various characteristics. To retain a realistic scale for the data values, we derived our simulated data from the Ovarian Cancer dataset (see "Materials and Methods"). The dataset was derived from 391 ovarian cancer patients in TCGA [21]. To obtain a baseline dataset with no signal, we randomly permuted the sample labels for each gene vector separately. Consequently, each gene vector retains its original distribution, but correlations between gene vectors are disrupted. We labelled *n *genes as active regulators in the simulated data. Each induces expression profiles in *p% *of its regulatee pairs that have a Pearson correlation coefficient of *r*. Regulators and regulatees are defined according to the literature-based causal network described earlier. To evaluate CSA with respect to many different signal-to-noise characteristics, we varied the percentage of correlated regulatee pairs *p *in 10% increments from 0% to 100%. Similarly, we set the correlation coefficient *r *to {0.3, 0.4, 0.5, 0.6}. Details on the generation of dependent profiles can be found in the Materials and Methods section.

To test the robustness of the method to sample size, we generated additional datasets with a random subset of 20, 100, and 200 patients. Finally, we generated simulated sets based on *n *= 10 as well as *n *= 100 embedded active regulators. We found that the recovery of true positives was not affected by the number of embedded regulators. We therefore fixed the number of embedded regulators in the subsequent examples at *n *= 100.

### Evaluation of permutation methods and controlling false positives

**Figure 3:**
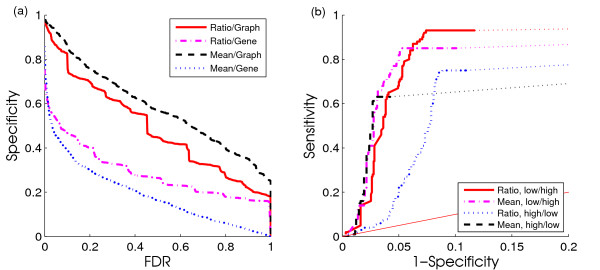
**False positive rates and ROC curves of CSA for different parameter settings**. (a) False positive rate of CSA with different parameter settings at different FDR levels. (b) ROC curves of mean function and ratio function of low/high and high/low simulated data. (a) The plot clearly shows that the estimated FDR can well control false positive rate of CSA. Both scoring functions with graph permutation reach low false positive rate when applying a reasonable FDR cutoff (FDR < 0.05). (b) The ROC curves suggests that the ratio scoring function reaches better true positive rate at the expense of a similar gain in false positive rate on datasets that contain few highly correlated regulatees.

While the scoring functions are able to rank embedded active regulators higher than non-active ones, they do not provide an objective cutoff value when investigating the biological significance of top results. In order to assess the suitability of our procedures to control the FDR, Figure 3a shows the false positive rate (for definitions, see "Materials and Methods") of the two scoring functions with the two permutation methods on a representative simulated data set (*r *= 0.5; *p *= 50%). Our procedures are able to control the false positive rate effectively based on the estimated FDR while retaining a good true positive rate (Additional file 1: Figure S1). In fact, when the data contains no or limited signal, CSA does not report any potentially active regulators at reasonable FDR cutoffs (FDR < 0.05). The same holds true for runs on randomized networks (data not shown).

Figure 3a also illustrates that *Graph permutation *is preferable to *Gene permutation*. ROC curves of Graph permutation and Gene permutation further prove that both graph permutation and gene permutation can reach good sensitivity and specificity, but graph permutation has higher specificity than gene permutation (Additional file 2: Figure S2). The purpose of our method is to find active regulators in a certain condition, which means that specificity might be more important than sensitivity in our case. We will only focus on *Graph permutation *results in the following. In contrast, the *mean *and *ratio *scoring functions seem to perform comparably, and a more in-depth analysis is needed.

### Evaluation of scoring functions

To understand the characteristics of the *ratio *and *mean *scoring functions, we focused on four datasets which differed in the strength of correlation r and the quantity of correlated regulatees p, namely, low/low (*r *= 0.3; *p *= 30%), low/high (*r *= 0.3; *p *= 80%), high/low (*r *= 0.6; *p *= 30%), high/high (*r *= 0.6; *p *= 80%).

Figure 3b depicts receiver-operator characteristic (ROC) curves (see "Materials and Methods" for definitions) for the low/high and high/low datasets. The only substantial difference between the two functions becomes apparent in the case of few highly correlated regulatee pairs, in which the *ratio *function is able to reach higher true positive rate at the expense of a similar loss in true negative rate. Note that the *ratio *function is explicitly designed to address this case. In most other situations, the two functions are comparable with the *mean *function performing slightly better. The results on all four datasets with respect to other performance metrics are shown in the supplementary materials.

### Robustness to signal level and sample size

**Figure 4:**
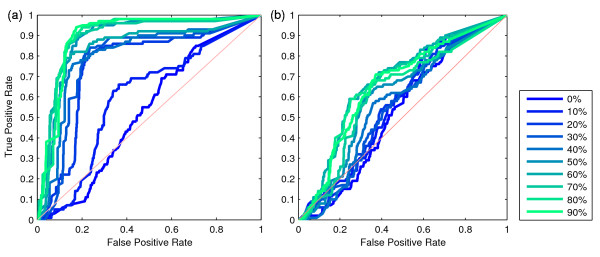
**Robustness of CSA with respect to different levels of signal (0%-90%) and sample size**. ROC curves based on (a) 391 samples and, (b) 20 samples. CSA reaches high true positive rate and low false positive rate for different signal levels.

Figure 4 shows the ROC curves under a variety of signal levels to demonstrate the ability of CSA to detect active regulators. In this instance, we use the *ratio *scoring function, but curves based on the *mean *function give similar results (data not shown). The curves demonstrate that CSA is able to pick out true active regulators embedded in the simulated data. For large sample sizes, the true positive and true negative rates were consistently high (> 80%) for a wide range of score cutoffs. As expected, performance deteriorated with decreasing signal, but remained useful, even for very low levels of signal (Figure 4a). In contrast, Figure 4b depicts the situation with only 20 patient samples. While for strong signals (*p > 70%*), some regulators can be detected, weaker signals result in performance close to random. Together, this shows that our causal network based on literature information is informative enough to enable recovery of embedded signals, given enough patient samples.

### Relevance of the causal network

To further illustrate the relevance of the underlying causal network to provide informative active regulators, we generated a randomized version of the causal network with the same degree distribution (using the edge-switching procedure described in the "Materials and Methods"). Running CSA based on this randomized network against the simulated data and ovarian cancer data from TCGA resulted in ROC curves that were indistinguishable from random, indicating that the causal network is biologically informative (data not shown).

### Comparison to degree-based ranking

**Figure 5:**
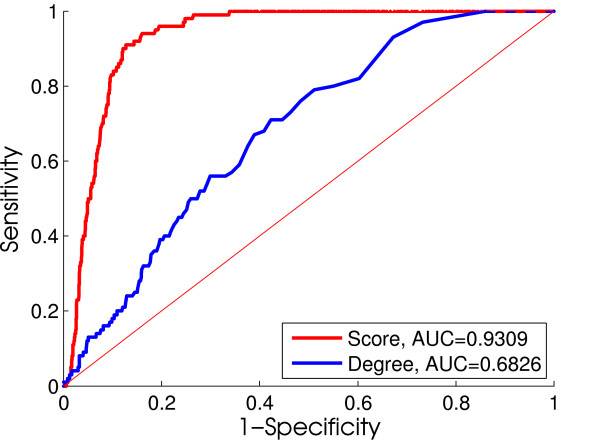
**ROC curves of *ratio *score ranking and degree-based ranking**. Red and blue ROC curves show ranking by *ratio *score and by out-degree (e.g. number of coherent expressed targets), respectively. The ROC curves suggest that degree-based ranking is better than random. However, ranking based on *ratio *score is superior to purely degree-based ranking.

Finally, we compare CSA's results to an alternative approach that has been suggested as a general principle in many approaches to transcriptional network reconstruction, namely the prediction of key regulators or biomarkers based on their degree in the inferred network [3,28,29]. Here, we use the same representative simulation data set (*r *= 0.5; *p *= 50%) as we used in the previous section. Implementing a ranking strategy based on each candidate regulator's out-degree (number of targets they coherently regulate) gives an interesting baseline performance (Figure 5) that is clearly better than random. However, the ROC curves suggest also that the results based on our method are superior to a purely degree-based method.

### Results on clinical data

To illustrate the utility of the CSA approach we describe here the results of comparing the directed perturbation experiments captured in the causal network to 3 different surveys of expression variation in 3 distinct disease and tissue settings-subcutaneous adipose tissue [22], ovarian cancer [21] and diffuse large B cell lymphoma [23].

### Experimental results I--adipose tissue

The first population dataset was subcutaneous adipose tissue from 673 individuals as described by Emilsson *et al. *[22], representing individuals from 3 generation families with a range of ages and degrees of obesity that was used to define loci affecting obesity in the Icelandic population. After matching transcripts measured in the adipose cohort to the causal network, the CSA method reported 246 of 1,762 (14%) regulators as potentially active at an FDR < 0.05. This corresponds to 8,946 potential regulator:regulatee edges.

These data can be summarized by counting the number of CSA significant regulatees for each regulator (see Additional file 3: Table S1). Amongst the top ranked regulators in adipose were some well-known metabolic targets, including PPARG (*n_c _*= 275), PPARA (*n_c _*= 218), Insulin (*n_c _*= 136) and PPARGC1A (*n_c _*= 105). The top hit as judged by the size of significant regulatees was MYC (*n_c _*= 391) which has been implicated in adipogenesis [30]. It is interesting to note that the well-known transcription factor, MYC, was not co-expressed with its regulatees in the adipose tissue dataset (average correlation coefficient = 0.1161), but a subset of its regulatees were coherently expressed. This observation supported our hypothesis. An additional top hit was, NFE2L2 (also known as NRF2, *n_c _*= 285), a master regulator of anti-oxidant response that has been implicated in many disease processes and in adipogenesis and obesity specifically [31].

**Figure 6:**
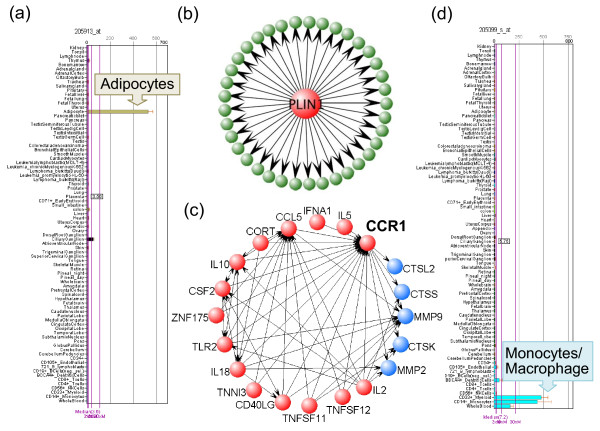
**CSA identifies important regulators expressed in adipocytes and monocytes**. (a) Gene expression of PLIN1 in different tissues. (b) PLIN1 and some of its downstream regulatees. (c) CCR1 and its upstream regulators (red nodes) and downstream regulatees (blue nodes). (d) Gene expression of CCR1 in different tissues. (a) and (d) are from BioGPS, which show that PLIN1 and CCR1 are uniquely expressed in adipocytes and Macrophages, respectively. (b) PLIN1 regulates 37 regulatees in adipose tissue. (c) CCR1 is regulated by numerous regulators in the causal network. CSA identified 14 potential active regulators of CCR1 in the adipose tissue (red). CCR1 is a regulator that can regulate several downstream regulatees (blue); at the same time, CCR1 is also regulated by many other regulators. These regulators regulate each other and also CCR1's regulatees.

Adipose tissue is composed of adipocytes and a stromal fraction including macrophages. Given this knowledge, we asked if CSA provided evidence for these sub-populations of cells. Perilipin (PLIN1) is a protein uniquely expressed in adipocytes (see Figure 6a and 6b) where it coats the surface of intracellular lipid droplets and protects them from degradation by lipases. CSA identifies 37 PLIN1 regulatees as cohesive in adipose tissue consistent with it being a significant regulator in human adipose tissue. A major conclusion of the adipose tissue study used here was that macrophages, as observed by macrophage-specific transcripts, are identified as causal drivers of obesity in humans [22] and mouse [32]. Consistent with this CSA finds a number of macrophage specific genes as active drivers including the chemokine receptor CCR1 (Figure 6c and 6d). One of the ligands of CCR1, RANTES is reported to be secreted by adipocytes and recruits macrophages to fat depots [33]. CCR1 appears as both a target of other regulators and as a regulator of downstream transcripts as judged by CSA (see Figure 6c). Furthermore the regulators of CCR1 were also found to be connected to each other consistent with a web of regulatory interactions affecting CCR1 and its downstream targets in macrophages in human adipose tissue.

Given the relative ease of experimentation, it is not surprising that many experiments reported in the literature were performed in cultured cell models. In this setting there is always a question of the relevance of the results to human populations. CSA potentially provides a data-driven way to assess this by testing whether any perturbation signature (or fraction of a signature) is significantly cohesive in a disease population. Interestingly, CSA identified many cell culture derived signatures as being relevant to the subcutaneous adipose tissue of humans (more than 53% individual references using cells in culture covered 47% regulator:regulatee edges). This included, for example, 88 of 254 regulatees for the classic adipose regulator PPARG and 68 of 116 insulin regulatees.

### Experimental results II--serous ovarian cancer

The CSA method was also assessed against a collection of expression profiles from almost 400 human serous ovarian cancers available through TCGA project [21]. After matching transcripts measured in the ovarian cohort with the causal network, it was found that 358 of 1,398 (25.6%) regulators had an FDR < 0.05 in the CSA analysis (see Additional file 4: Table S2). This identifies 12,860 potential regulator:regulatee edges as active in this cohort.

**Figure 7:**
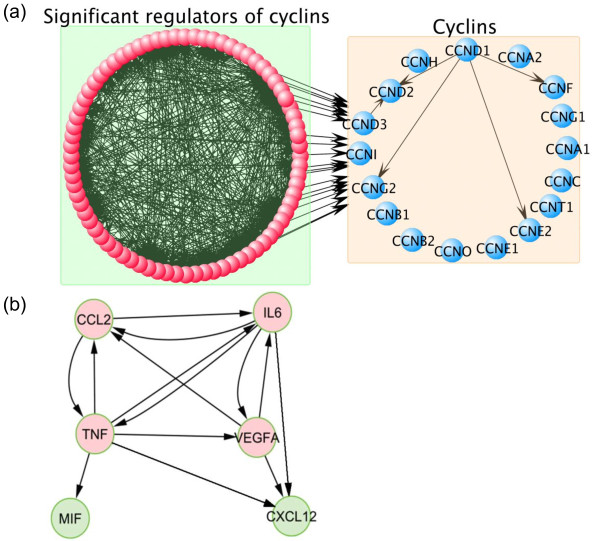
**Important regulators and hypothetical regulatory model in serous ovarian cancer**. (a) Regulators regulate cyclins in serous ovarian cancer. (b) Hypothesis regulatory model of secreted proteins in serous ovarian cancer. (a) Cyclins have 603 regulators in the causal network. CSA identified 358 potential active regulators in serous ovarian cancer; 77 of 358 regulators were found to regulate cyclins. Regulators (red nodes) regulated cyclins (blue nodes) and also regulated each other, which implies that these regulators work cooperatively to regulate cyclins. (b) Secreted proteins TNF, IL6, VEGFA and CCL2 were identified as regulators (red nodes) in serous ovarian cancer by CSA. They regulated each other and two other secreted proteins, MIF and CXCL12 (green nodes). TNF, IL6, VEGFA and CCL2 are also used as therapeutic targets of several different kinds of cancers [35-38].

As before, the potential regulators can be ranked by the number of regulatees that pass the cutoff. Top ranked regulators include genes implicated in many cancers (see Additional file 4: Table S2) such as TGFB1 (*n_c _*= 520), IFNG (*n_c _*= 485), FGF2 (*n_c _*= 241), MYCN (*n_c _*= 219) and VEGFA (*n_c _*= 183). Again, we observed that another well-known transcription factor, MYCN, was weakly correlated with its regulatees (average correlation coefficient = 0.1015), but it had a subset of coherent regulatees and were identified by CSA as active in ovarian cancer dataset. CSA also identifies numerous potential drivers of various cyclins which are known to drive the cell cycle and be aberrantly regulated in cancer (see Figure 7a). Among 603 regulators that regulate cyclins in the causal network, CSA identified 77 as active in the serous ovarian cancer. As with the CCR1 example extensive cross-regulation of cyclin regulators was predicted, suggesting the presence of a complex network of causal interactions upstream of cyclins in ovarian cancers revealed by CSA. The derivation of the higher-order structure is non-obvious given the literature examples alone, but readily emerges from the CSA analysis as relevant in this context. Using the ranking by the number of edges testing significant in CSA, TNF was found to have 657 regulatees (reactive to TNF) and 153 regulators (causal regulators of TNF) in this cohort. High levels of secreted TNF protein (ranked first by CSA) were reported to cause high levels of the proteins IL6, MIF, CCL2, CXCL12, VEGFA, also secreted in ovarian cancer cell lines [34]. Intriguingly, these genes in the CSA analysis of the ovarian cancer cohort were also found to have many regulatees (304, 0, 13, 0, 183, respectively) and/or regulators (158, 5, 64, 18, 26, respectively), and directly and reciprocally regulate each other (see Figure 7b). As reported by Kulbe *et al. *[34], knockdown of TNF in cells with high TNF results in failure to form tumors in mice. The interpretation of such established cell line models of cancer can be difficult. The CSA analysis however indicates that the basic regulatory findings of this work were replicated by CSA and found to be the most dominant feature in approximately 400 ovarian clinical cancer samples. Together, these findings may identify a critical driver of ovarian tumor growth *in vivo*.

### Experimental results III--DLBCL

**Figure 8:**
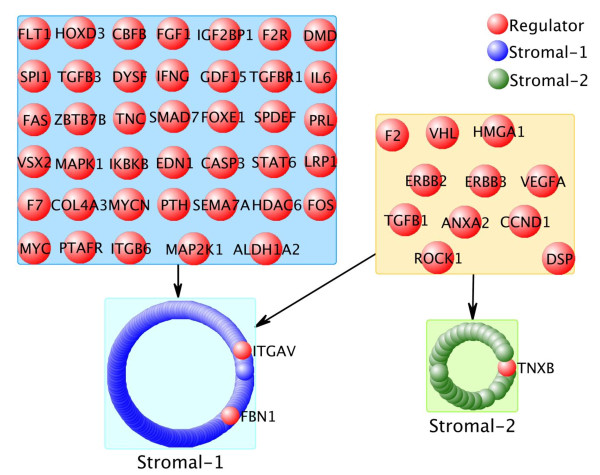
**Regulators enriched for Stromal-1 and Stromal-2 signatures**. Red nodes are regulators. Blue nodes are genes in Stromal-1 signatures. Green nodes are genes in Stromal-2 signatures. We did not find any regulator enriched for Germinal Center B-cell signature works in both cohorts. Instead, CSA identified 55 and 11 regulators enriched for Stromal-1 and -2 signatures in both cohorts. Furthermore, the regulatory model showed that the majority of genes in Stromal-2 signature are regulated by regulators that also regulate Stromal-1 signature.

The final example is a study of diffuse large B-cell lymphoma (DLBCL) in which expression profiles of 2 patient populations who subsequently received different treatments were examined for signatures that predict the clinical course of the disease [23]. For the purposes of this analysis the subsequent treatments are not relevant. The first cohort (CHOP) included 181 samples and the second cohort (R-CHOP) included 233 samples. As described in [23], 3 signatures were derived in a multivariate analysis that predict survival in the 2 cohorts. The *Germinal Center B-cell *signatures contained 37 genes, the *Stromal-1 *signature contained 264 genes and the *Stromal-2 *signature contained 61 genes. CSA analysis was applied to each of the cohorts and potentially active regulators identified that pass the FDR cut-off (218 and 220 of 1780 significant hits for CHOP and R-CHOP, respectively (see Additional file 5: Table S3). Using these significant hits we then asked if any of the regulators regulated genes involved in the 3 predictive signatures (*Germinal Center B-cell, Stromal-1 *or *Stromal-2*). Interestingly, although the *Stromal-1*, and -*2 *signatures were found by a multivariate analysis, suggesting they are independent, CSA analysis identifies genes that can regulate both signatures jointly. Among the 131 regulators that regulate at least one gene in either the *Stromal-1 *or -*2 *signatures, 53 (40%) regulate genes in both cohorts. Furthermore, we calculated the significance of the enrichment of each regulator's regulatees for overlap with the 3 predictive signatures by Fisher's exact test. Significant enrichments for the two *Stromal *signatures were found (see Table 1). Figure 8 shows the regulators enriched for *Stromal-1 *and -*2 *signatures in the CHOP and R-CHOP cohorts and their target genes in all three signatures. 11 regulators were found enriched for *Stromal-2 *signature in both cohorts. Surprisingly, all of these 11 regulators are also enriched for *Stromal-1 *signature, indicating that it is possible the 2 signatures arise because of the same regulator(s).

**Table 1:** Top 15 regulators found in CHOP and R-CHOP cohorts

	**No. of Regulatees**^**a**^		**Germinal Center B cell**^**b**^		**Stromal-1**^**c**^		**Stromal-2**^**d**^	
Regulator	CHOP	R-CHOP	CHOP	R-CHOP	CHOP	R-CHOP	CHOP	R-CHOP
TGFB1	723	632	0.2379	0.3081	**1.36E-12**	**2.30E-14**	**0.0003**	**7.36E-05**
IFNG	624	553	0.5868	0.6600	**0.0041**	**0.0006**	0.1928	0.1008
MYC	439	410	0.1870	0.2094	**0.0013**	**0.0005**	0.1427	0.0673
IL6	370	317	0.5962	0.6678	**0.0281**	**0.0497**	0.2135	0.0854
MYCN	272	248	0.3566	0.3908	**8.22E-06**	**7.20E-06**	0.4979	0.5463
ERBB2	253	219	0.3731	0.3666	**7.50E-07**	**2.79E-07**	**0.0149**	**0.0088**
IFNB1	251	233	0.3864	0.4139	0.1869	0.1256	0.4674	0.4826
IL10	249	221	0.3727	0.3672	0.0950	0.0543	0.1307	0.1080
CDKN1A	234	224	0.3704	0.3680	0.0953	0.0554	0.4817	0.2436
F2	233	212	0.4139	0.4484	**0.0009**	**0.000372**	**0.0409**	**0.0317**
IL2	205	188	0.4605	0.4912	0.3236	0.4066	0.6388	0.6769
CCND1	204	184	0.4622	0.4987	**7.86E-05**	**2.38E-05**	**0.0013**	**0.000777**
VEGFA	189	170	0.4894	0.5259	**3.27E-05**	**3.60E-05**	**1.86E-06**	**7.91E-06**
MAPK1	183	166	0.5006	0.5340	**0.0063**	**0.003266**	0.3192	0.3550
TNFSF11	166	150	0.1038	0.0898	0.5435	0.6276	0.1905	0.1714

^a^number of cohesive regulatees.

^b, c, d^the enrichment p-values of Germinal Center B cell, Stromal-1 and Stromal-2 signatures in CHOP and R-CHOP groups.

The candidate regulators can be ranked by the number of predictive signature genes they regulate (limiting to those enriched for the signatures). This results in the identification of some very familiar drivers of many cancers including MYC, MYCN and CCND1 (see Table 1).

### Results on clinical data with the public causal network

Selventa Inc. has recently launched an initiative to provide access to a significant amount of causal information to academic researchers. We performed CSA on the ovarian cancer dataset with the public causal network released by Selventa Inc. The result suggested that 121 of 170 regulators reported by CSA (FDR < 0.05) with the public causal network were found in our previous result (Additional file 6: Table S4). Compared with 358 causal regulators identified by using the complete causal network, CSA can recover about 1/3 of the regulators in the ovarian cancer dataset. The results suggested that CSA works well with the public causal network although it does not report as many causal regulators as with the complete causal network.

## Conclusions

The advent of inexpensive high-throughput transcriptomics measurement techniques has enabled the characterization of cell lines, animal models, and, more recently, cohorts of clinical patients on a molecular level. A crucial research question in such studies (e.g. in ovarian cancer patients) is the identification of causal regulators of the observed transcript changes. In this study, we sought to develop a method, Correlation Set Analysis (CSA), to identify directed perturbation experiments relevant to a disease population of interest in an unbiased data-driven manner. The method relies on the hypothesis that regulatees of a regulator active in a population will be significantly correlated to each other across the population. As described the CSA method was developed and evaluated on simulated data where it found to be quite sensitive in identifying known regulator effects.

To test the method and illustrate its uses, we present the results of CSA analysis in three disease settings, where in each case known key regulators and as yet un-described regulators were found. In the regulatory networks of CCR1 in adipose tissue and cyclins in serous ovarian cancer, we observed that upstream regulators of CCR1 and cyclins not only regulated CCR1 and cyclins but also regulated each other. Also in the DLBCL dataset, we found that of 11 regulators enriched for Stromal-2 signature in both CHOP and RCHOP cohorts were also enriched for Stromal-1 signature. This finding implies that Stromal-1 and -2 signatures are actually regulated by the same regulators. These examples indicate that CSA is able to bring together isolated regulatory relationships into higher order regulatory networks, and thereby adding new knowledge and insights.

Based on this we propose that CSA has four key advantages. Firstly, it provides a simple data driven way to connect population data with the many directed perturbation experiments reported in the literature. Secondly, CSA identifies plausible changes in regulators at the protein level, since we do not require correlation between regulator and regulatees. A third advantage of this approach is that the results can be simply ranked providing the user with an intuitive way to prioritize any follow up investigation. Finally, the regulatory relationships inferred by CSA were found in all cases to form a contiguous network, with many cases of regulators that were also regulatees of upstream regulators. In other words CSA provides a glimpse of the true underlying higher order structure of biological systems which can be systematically tested for causal effects on disease progression.

In addition to the advantages it is worthwhile noting that there are some limitations to CSA that stem from its reliance on the literature-derived causal network. Clearly, the relationships identified are limited to the causal network used. With the continued expansion of our understanding of regulatory relations, the accuracy of CSA should improve with time. Another possible limitation is in cases where overlapping sets of regulatees are controlled by distinct regulators. In this case, CSA will not be able to distinguish between the regulators resulting in an increased false positive rate. One possible solution for this problem is to cluster regulators by the similarity of their respective regulatee sets. In our experience, however, the overlap between regulatee sets is still sufficiently small to allow for meaningful inference. This is supported by the excellent performance of CSA on simulated data.

As we mentioned before, the causal network in its original form contains not only genes and proteins, but also chemical compounds and environmental factors. Since our approach does not restrict regulators to genes on the microarray, such entities can appear as candidate regulators, though we did not report them in this work. Hypotheses based on compounds might be useful for assessing environmental factors driving disease progression or for supporting drug repositioning efforts.

In the future we plan to apply the CSA method to large scale compendia of gene expression sets to understand patterns of active regulation across a span of phenotypes. Given the promising performance of CSA when recovering embedded regulators as well as providing insights for biological datasets, we believe that it will greatly enhance our understanding of general and specific mechanisms driving disease and other relevant phenotypes.

## Materials and methods

### Generating simulated data

In order to make simulated data follow the distribution of the real data, we generate the simulated data based on the ovarian cancer data used in this study by applying the Cholesky decomposition method of Iman and Conover [39]. This approach is widely used to generate correlated random variables in finance. First, we randomly permute sample labels of a gene and repeat this step for all genes. Hence, the mean and standard deviation of each gene vector are invariant under permutation. The correlation between genes, however, is disrupted. Then, we randomly pick N regulators as test set of regulators. Each test regulator *R_p _*can regulate a set of regulatees *T *= {*T*_1_...*T_q_*}, *T *is an *n *× *m *matrix, where *n *is the number of regulatees and *m *is the number of samples (*m *= 391 in this case). We want to generate a set of new regulatees $T^{s}=\left\{T_{1}^{s}\ldots T_{q}^{s}\right\}$with same mean and variance as the original set of regulatees but correlate with each other at a desired correlation coefficient *ρ*. An *n *× *n *covariance matrix ∑ s with desired correlation coefficient *ρ *is created. The ∑ must be symmetric and positive-semidefinite. Therefore, it can be rewritten as ∑ = *LL*' by Cholesky decomposition, where *L *is the lower triangular matrix of ∑ with positive diagonal entries. We can get "spike-in" regulatee matrix *T*^s ^with desired correlation coefficients *ρ *by postmultiplies *T *by *L*. Iterate above steps until all regulatees of the test regulators are modified to be correlated with correlation coefficient *ρ*.

### Experimental data

Expression datasets of adipose tissue and DLBCL are downloaded from the Gene Expression Omnibus [40]. Adipose tissue samples from 701 individuals [GEO:GSE7965] with a range of age from 18 to 85 and average BMI nearly 30 were used in this study. Pretreatment tumor samples from 181 and 233 DLBCL patients [GEO:GSE10846] were used in this study. TCGA [1] provides mRNA measurements of serous ovarian cancer tissue using 3 array platforms: an Agilent array, and Affymetrix's U133A and exon arrays. Where genes are overlapping among the 3 platforms, we would like to combine the values into a consensus gene. Here, we follow an approach originally described by Verhaak *et al. *[41]. In short, the consensus gene is estimated using a standard factor model based approach:

$$\hat{x}={\left({\hat{\beta }}^{T}{\left(\hat{\beta }{\hat{\beta }}^{T}+\hat{\Psi }\right)}^{-1}y\right)}^{T}$$

where $\hat{\beta }$and $\hat{\Psi }$are the platform specific coefficients and error covariance estimates, respectively, *y *is the 3-by-*m *dimensional gene expression values across the 3 platforms, and $\hat{x}$is the *m*-dimensional, unified estimate for a single gene. For complete details, see Verhaak, *et al. *[41]. In those cases where only 2 genes are shared across the 3 platforms, we take the mean value.

For all of the 3 datasets, we used LSimpute to impute missing values in the expression profiles [42]. We discarded genes that are not included in our causal network and leave 9,052, 9,950 and 7,673 genes in adipose tissue dataset, DLBCL dataset and ovarian cancer dataset respectively.

### Performance Metrics

The receiver operating characteristic (ROC) curves are used to evaluate the performance of CSA. The true positive rate and false positive rate used for plotting ROC curves are calculated as following:

$$\text{True positive rate}=\frac{\text{True positives}}{\text{True positives}+\text{False negatives}}$$

$$\text{False positive rate}=\frac{\text{False positives}}{\text{False positives}+\text{True negatives}}$$

### Graph permutation

The corresponding permutation scheme (*Graph permutation*) is more complex and also computationally more intensive. In each permutation, we evaluate S_R _on a random graph with the same degree distribution as our causal network. Randomizing a directed graph with a given degree sequence is an active field of research and we adopt a method from [43] relying on edge switching.

More precisely, at every iteration we pick two edges, say (a, b) and (c, d), uniformly at random from the set of edges E in the current graph, and replace them with the edges (a, d) and (c, b). This operation is known as an edge switch, and preserves the in- and out-degree distribution of the graph. If the resulting graph remains simple (no parallel edges) and weakly connected, it replaces the current graph. In order to save the computationally expensive connectivity checks, a batch of K edge switches can be performed before a connectivity check. If the check succeeds, K can be increased, while if it fails, K can be decreased. The particular adaptive algorithm we use to update K is described in [43]. We also adopt the commonly used rule of thumb [44] for the total number of edge switches to perform before declaring our graph to be sufficiently randomized, which is to perform an average of 3 edge switches per edge of the initial graph. Both permutation approaches assert the statistical significance of a score S_R _under the respective null hypothesis, and thus provide guidance to the biologists as to whether a particular regulator received a high score based on chance alone.

## Abbreviations

CSA: Correlation set analysis; FDR: False discovery rate; TCGA: The cancer genome atlas; DLBCL: Diffuse large B-cell lymphoma; ROC: Receiver-operator characteristic.

## Competing interests

The authors declare that they have no competing interests.

## Authors' contributions

CLH and DZ designed the research and developed the method. CLH implemented and tested the method. DZ, CLH and JL drafted the manuscript. JL interpreted the results of experimental datasets. LC performed graph permutation and helped in the writing of the manuscript. JG carried out the integration of expression data of ovarian cancer and helped in the writing of the materials section. JK provided suggestions for the statistical assessments. CD provided initial guidance. DZ, JL, LC and CD edited the manuscript. All authors read and approved the final manuscript.

## Supplementary Material

Additional file 1 **Figure S1 Effects of permutation on ranking regulators**. ROC curves show that FDR calculated based on permutation can improve both sensitivity and specificity. Graph permutation is used here. Permutation and FDR correction can decrease false positives by filtering out regulators that have high scores but only regulate a small number of regulatees since a regulator can easily obtain a high score by chance if it only regulates a few regulatees. Similarly, permutation and FDR correction can increase true positives by recruiting regulators that have fair scores but can regulate a large number of regulatees. Click here for file

Additional file 2 **Figure S2 Comparison of graph permutation and gene permutation on two representative simulation data sets**. (a) Simulation data set (r = 0.5, p = 50%). (b) Simulation data set (r = 0.3, p = 70%). Scoring function "ratio" is used in both cases. Both permutation methods can reach good sensitivity and specificity. However, graph permutation reaches slightly better specificity in most cases. Click here for file

Additional file 3 **Table S1 Active regulators found in the adipose tissue**. Click here for file

Additional file 4 **Table S2 Active regulators found in the serous ovarian cancer**. Click here for file

Additional file 5 **Table S3 Active regulators found in the DLBCL**. Click here for file

Additional file 6 **Table S4 Active regulators found in the serous ovarian cancer when using data from Selventa's BEL portal**. Click here for file
