# Supplementary figures and images for: Correlation set analysis: detecting active regulators in disease populations using prior causal knowledge
Source: BMC Bioinformatics. 2012 Mar 23;13:46. doi: 10.1186/1471-2105-13-46 (PMC3382432; doi:10.1186/1471-2105-13-46)

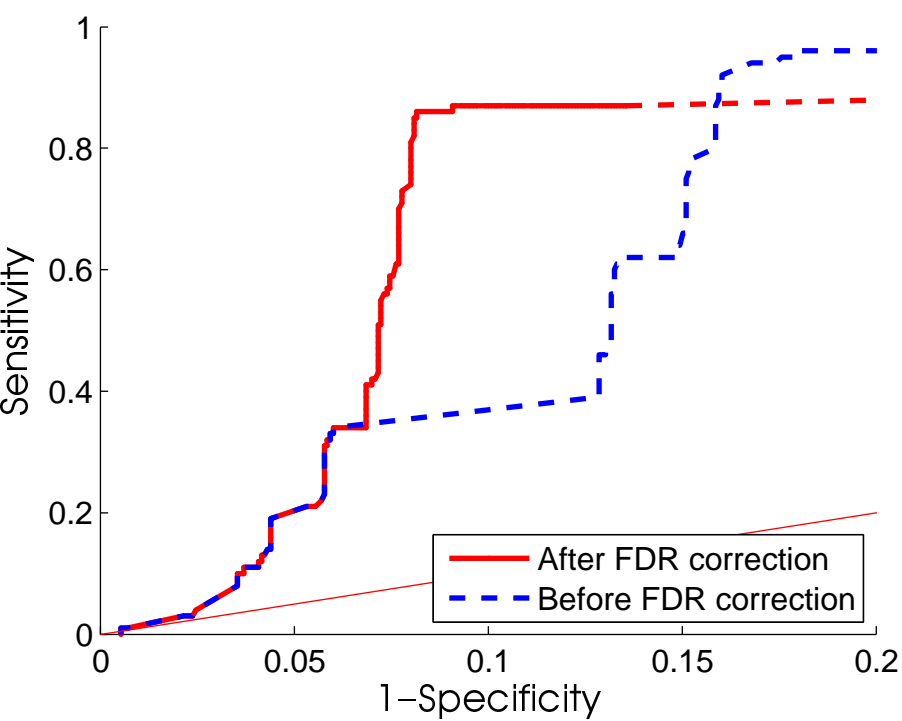

Supplement: Additional file 1 — Figure S1 Effects of permutation on ranking regulators. ROC curves show that FDR calculated based on permutation can improve both sensitivity and specificity. Graph permutation is used here. Permutation and FDR correction can decrease false positives by filtering out regulators that have high scores but only regulate a small number of regulatees since a regulator can easily obtain a high score by chance if it only regulates a few regulatees. Similarly, permutation and FDR correction can increase true positives by recruiting regulators that have fair scores but can regulate a large number of regulatees. [file 1471-2105-13-46-S1.PDF]

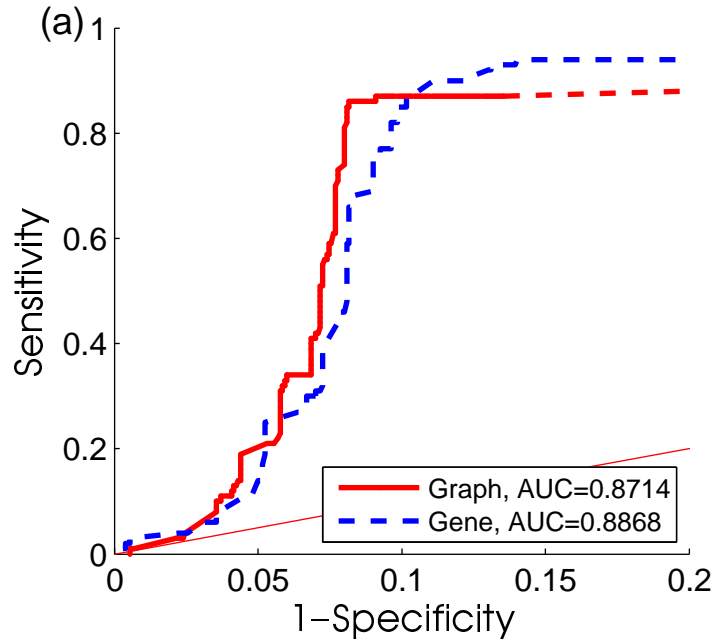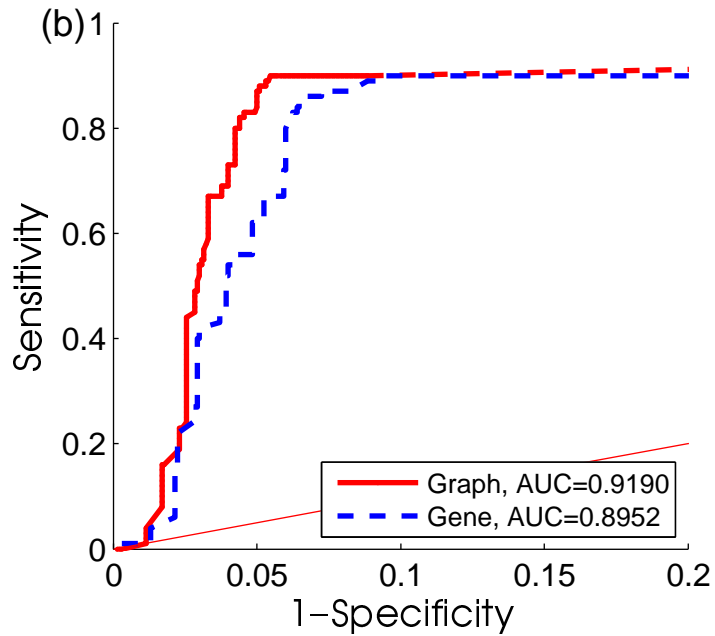

Supplement: Additional file 2 — Figure S2 Comparison of graph permutation and gene permutation on two representative simulation data sets. (a) Simulation data set (r = 0.5, p = 50%). (b) Simulation data set (r = 0.3, p = 70%). Scoring function "ratio" is used in both cases. Both permutation methods can reach good sensitivity and specificity. However, graph permutation reaches slightly better specificity in most cases. [file 1471-2105-13-46-S2.PDF]
